# A Wearable Device Based on a Fiber Bragg Grating Sensor for Low Back Movements Monitoring †

**DOI:** 10.3390/s20143825

**Published:** 2020-07-09

**Authors:** Martina Zaltieri, Carlo Massaroni, Daniela Lo Presti, Marco Bravi, Riccardo Sabbadini, Sandra Miccinilli, Silvia Sterzi, Domenico Formica, Emiliano Schena

**Affiliations:** 1 Department of Engineering, Università Campus Bio-Medico di Roma, Via Alvaro del Portillo, 00128 Rome, Italy; m.zaltieri@unicampus.it (M.Z.); c.massaroni@unicampus.it (C.M.); d.lopresti@unicampus.it (D.L.P.); riccardo.sabbadini@ieee.org (R.S.); 2 Unit of Physical Medicine and Rehabilitation, Università Campus Bio-Medico di Roma, Via Alvaro del Portillo, 00128 Rome, Italy; m.bravi@unicampus.it (M.B.); s.miccinilli@unicampus.it (S.M.); s.sterzi@unicampus.it (S.S.); 3 Unit of Neurophysiology and Neuroengineering of HumanTechnology Interaction, Università Campus Bio-Medico di Roma, Via Alvaro del Portillo, 00128 Rome, Italy; d.formica@unicampus.it

**Keywords:** Fiber Bragg Grating sensors, wearable devices, low back pain, occupational safety, video terminal workers

## Abstract

Low back pain (LBP) is one of the musculoskeletal disorders that most affects workers. Among others, one of the working categories which mainly experiences such disease are video terminal workers. As it causes exploitation of the National Health Service and absenteeism in workplaces, LBP constitutes a relevant socio-economic burden. In such a scenario, a prompt detection of wrong seating postures can be useful to prevent the occurrence of this disorder. To date, many tools capable of monitoring the spinal range of motions (ROMs) are marketed, but most of them are unusable in working environments due to their bulkiness, discomfort and invasiveness. In the last decades, fiber optic sensors have made their mark allowing the creation of light and compact wearable systems. In this study, a novel wearable device embedding a Fiber Bragg Grating sensor for the detection of lumbar flexion-extensions (F/E) in seated subjects is proposed. At first, the manufacturing process of the sensing element was shown together with its mechanical characterization, that shows linear response to strain with a high correlation coefficient (R^2^ > 0.99) and a sensitivity value (S_ε_) of 0.20 nm∙mε^−1^. Then, the capability of the wearable device in measuring F/E in the sagittal body plane was experimentally assessed on a small population of volunteers, using a Motion Capture system (MoCap) as gold standard showing good ability of the system to match the lumbar F/E trend in time. Additionally, the lumbar ROMs were evaluated in terms of intervertebral lumbar distances (Δ$d_{L3-L1}$) and angles, exhibiting moderate to good agreement with the MoCap outputs (the maximum Mean Absolute Error obtained is ~16% in detecting ${\Delta d}_{L3-L1}$). The proposed wearable device is the first attempt for the development of FBG-based wearable systems for workers’ safety monitoring.

## 1. Introduction

In the last decades, the population of employees involved in computer working has been constantly growing since video terminals have become an essential tool in almost all work environments [1,2,3]. Video terminal workers (VDTs), especially keyboard users, experience prolonged static positions, unnatural and uncomfortable postures, as well as excessive proximity to screens for most of their working day [4]. Therefore, VDTs are often predisposed to visual disease (e.g., eye soreness) and musculoskeletal disorders (MSDs) such as low back pain (LBP) and neck pain, shoulder ache and wrist soreness [5]. Among these, LBP is one of the most common diseases occurring amongst VDTs, suffice it to say that in Europe about 44 million workers suffer from lumbar ache [6,7]. Causing a loss of 149 million working days per year in the US alone [8], LBP is considered to be one of the main reasons for absenteeism in workplaces, as well as a relevant socio-economic burden, since it leads to reduced work productivity, high insurance costs and the exploitation of the National Health Service [9,10]. Studies show that the occurrence of LBP is attributable to multiple factors that take into account both the workers’ physical features (i.e., age, gender, and body mass index—BMI) and the working environment [11]. Nevertheless, the adequacy of the working environment (that is, the adoption of ergonomic seating and comfortable workstations) is of paramount importance, although frequently not sufficient to avoid the onset of such a disease. In this context, being able to avert the occurrence of LBP by preventing VDTs from assuming incorrect postures is crucial. Such a goal can be achieved by examining employees’ spinal range of motions (ROMs). In fact, ROMs can be helpful to detect a range of intervertebral angles out of which the workers’ posture is deemed incorrect and unnatural. Traditionally, goniometers [12], radiographs [13,14] and motion capture systems (MoCap systems) [14,15] are the most exploited devices for lumbar ROMs detection in the medical practice. Although accurate, such systems prove to be cumbersome or uncomfortable to use during the whole working day. Therefore, to overcome these limits, the technology has increasingly moved towards the development of lightweight and wearable devices such as the ones embedding inertial sensors and piezoresistive textiles [16], strain gauge sensors [17] or fiber optic sensors (FOSs) [18,19,20]. In particular, in the last twenty years FOSs played a crucial role in the implementation of compact wearables able to monitor spinal ROMs thanks to the properties of such technology (i.e., flexibility, lightness, handling, and multiplexing capability [21,22,23]). Williams et al. [18] and Cloud et al. [19] equipped a commercial device (i.e., ShapeTape S128048NL, ShapeTape^TM^, Measurand, Fredericton, NB, Canada) made by a ribbon of sprung steel, with several arrays of FOSs. Although precise in detecting spinal ROMs, this device is required to be fixed on the employees’ skin making it unsuitable to be worn in workplaces. A different approach was used by Dunne et al. [20] who presented a comfortable commercial tight shirt integrated with FOSs. Among FOSs, Fiber Bragg Grating (FBGs) sensors have gained broad acceptance to instrument wearable systems. Several wearable devices embedding FBGs capable of monitoring cardiorespiratory parameters have already been proposed by our research group [24,25,26,27,28,29], but no FBG-based system for lumbar ROMs detection has been investigated yet. In the present work, a novel wearable device based on FBG technology for monitoring low back flexion/extension (F/E) movements is presented. Such wearable device is composed by a handcrafted elastic structure equipped with a flexible sensing element which is made by a soft silicone patch integrated with an FBG sensor. The device is comfortable, easily-wearable upon clothes and can be tailored to different body shapes. Firstly, the mechanical characterization of the sensitive element was performed. Then, the feasibility assessment of the system in detecting and following F/E lumbar movements was executed on four healthy volunteers performing F/E movements while sitting and wearing the proposed system. 

## 2. Design and Development of the Wearable System

### 2.1. Sensing Element Based on FBG

In the present work, a lightweight flexible sensor based on FBG technology was developed to monitor low back F/E movements. The manufacturing process and the sensor working principle are described in the following paragraphs.

#### 2.1.1. Design and Manufacturing

A rectangular-shaped plastic mold, whose dimensions are 55 mm × 20 mm × 2 mm, was created with Onshape^®^ design software and realized by the 3D printing process [30] (‘Ultimaker 2+’, Ultimaker B.V., Utrecht, The Netherlands). Then, the flexible sensing element was produced by encapsulating a commercial optical fiber embedding an FBG (grating length of 10 mm, Bragg wavelength—λ_B_—of 1556.997 nm and reflectivity of 90%; AtGrating Technologies, China) into a silicone substrate (Dragon Skin^TM^20, Smooth-On, Inc., Macungie, PA, USA). Such liquid rubber is a bi-component compound (Part A and Part B) that, once cured, constitutes a highly flexible and stretchable support base that improves the FBG in robustness avoiding breakages. 

**Figure 1:**
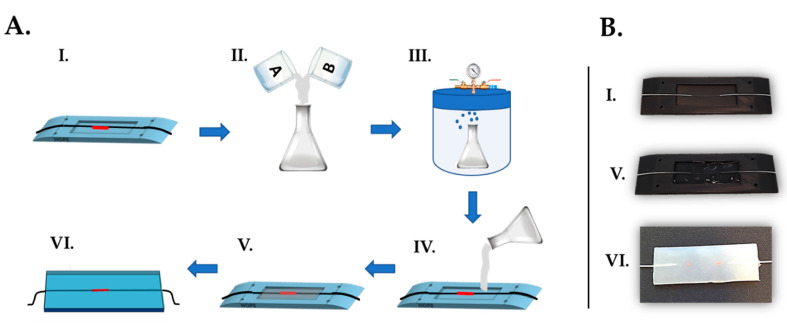
Manufacturing process of the flexible FBG-based sensing element. (**A**): Schematic of the fabrication steps; (**B**). Details of the phases I., V. and VI.

The flexible sensor manufacturing process (see Figure 1) consists of the steps illustrated below:

1. The FBG was placed at the midsection of the custom-made plastic mold. The extremities of the optical fiber were then passed inside the lateral grooves and delicately fixed with the help of some adhesive tape in order to keep the fiber adequately tight;
2. Dragon Skin^TM^20 silicone rubber parts A and B were mixed 1A:1B by volume ratio (as indicated in the technical bulletin [31]). Then, an amount of 10% in volume of liquid thinner was added to reduce the viscosity of the compound. The mixture was well stirred in order to allow the complete blending of all the components;
3. The compound was put into a vacuum chamber and let degas for few minutes in order to obtain an opalescent fluid with no presence of gas bubbles;
4. The degassed mixture was slowly poured into the mold until its full filling;
5. The mixture was let polymerize for a curing time of four hours at room temperature (as indicated in the technical bulletin [31]);
6. Once solidified, the flexible rectangular-shaped (i.e., 55 mm × 20 mm × 2 mm) sensing element was extracted from the mold. The excess of polymeric material was removed by means of a cutter and the edges were refined.

**Figure 2:**
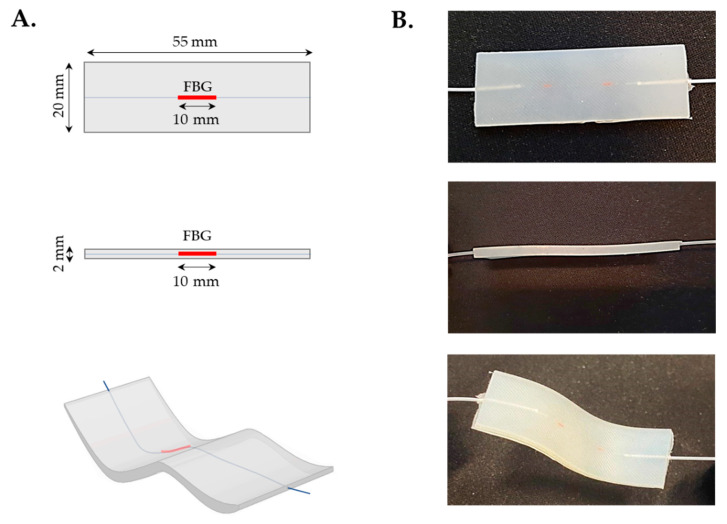
The flexible FBG-based sensing element. (**A**): Shape and size of the sensor in frontal view (up), lateral view (middle) and warped configuration (bottom); (**B**): Details of the sensor in frontal view (up), lateral view (middle) and warped configuration (bottom).

In Figure 2 the flexible sensing element is shown together with its features (i.e., shape and dimensions). 

**Figure 3:**
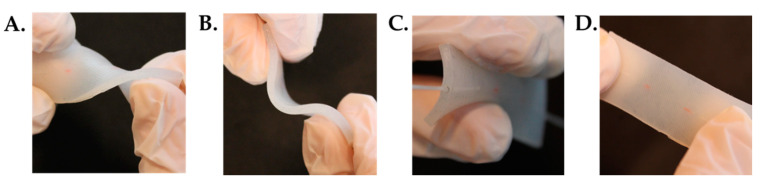
The flexible element in four different configurations: (**A**) twisting, (**B**) bending, (**C**) folding and (**D**) stretching.

The high flexibility of the sensor is exhibited in Figure 3, where the twisting, bending, folding and stretching capabilities of the element are shown. 

#### 2.1.2. Working Principle

An FBG is a periodic modulation of the effective refractive index of an optical fiber obtained by inscribing a small portion (typically from 3 to 20 mm) of fiber core with an intense ultraviolet (UV) source [32]. Once illuminated with a broadband light by means of a fiber optic interrogator, the FBG works as a stop band filter. In fact, most of the incident light is transmitted along the fiber, while a small portion of spectrum is reflected back to the interrogator. The reflected spectrum is set at λ_B_. Considering that η_eff_ is the fiber core effective refractive index and Λ is the grating period, λ_B_ can be defined as follows:

(1) $$\lambda_{B}=2\cdot \eta_{\mathrm{eff}}\cdot \Lambda$$

Both η_eff_ and Λ are strictly related to temperature variations (∆T) and strain (ε). As changes in ∆T and ε produce a λ_B_ shift (Δλ_B_), FBGs are considered to be excellent sensing elements to evaluate such parameters. The Δλ_B_ is described by the Equation (2):

(2) $$\frac{c}{\lambda_{B}}=\left(1-{\;p}_{e}\right)\cdot \varepsilon +\left(\left(1-{\;p}_{e}\right)\cdot \alpha +\;\xi \right)\cdot \Delta T$$

where p_e_ , α and ξ represents the effective photoelastic coefficient, the fiber thermal expansion coefficient and the fiber thermo-optic coefficient, respectively [33].

In the presented application, the stretching and bending effects caused on the silicone substrate by the F/E movements are transmitted to the FBG which is consequently subjected to ε deformations. In fact, at the end of each flexion, the sensor experiences the maximum elongation resulting in the highest Δλ_B_ value; on the contrary, at the end of each extension the sensitive element experiences the maximum contraction resulting in the lowest Δλ_B_ value. The ∆T contribute is considered to be negligible as demonstrated in a previous study [28]. Moreover, in this specific application, the experimental routines were performed at constant room temperature and the sensing element was placed upon the elastic structure, not in direct contact with the subjects’ skin. Therefore, the Δλ_B_ is mainly given by the ε contribute, and the sensing element can be considered as a strain sensor only. 

#### 2.1.3. Mechanical Characterization 

**Figure 4:**
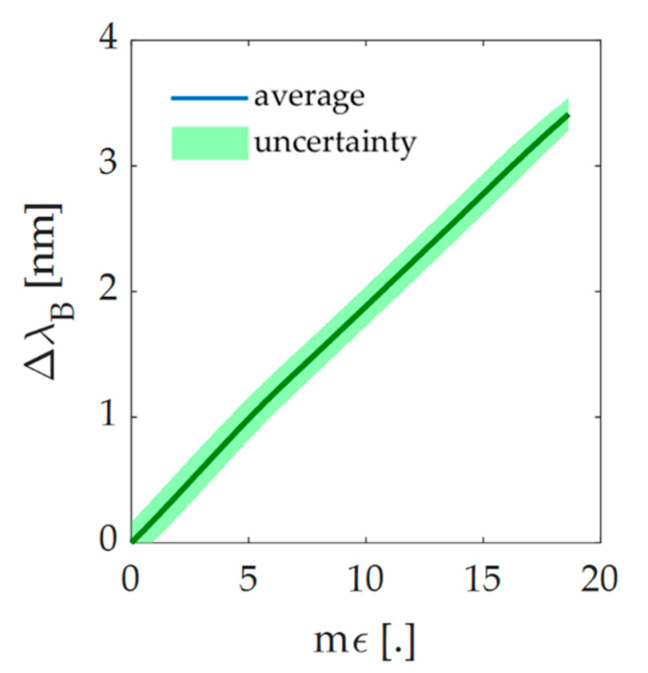
Response to strain of the sensing element. Trend of the calibration curve (blue curve) and its uncertainty (green shadow).

A set up constituted by a fiber optic interrogator (Micron Optics si255, Micron Optics Inc., Atlanta, GA, USA) and a tensile testing machine (Instron 3365A, Instron, Norwood, MA, USA) was used to estimate the response to strain of the flexible sensor. A tensile test was performed on the flexible sensor at room temperature and quasi-static conditions (i.e., low load speed). The sensitive element was secured to the two machine clampers, being careful to place the FBG in the middle. It was then lengthened at 2 mm·min^−1^ of load speed, by 2% with respect to its initial length. The point of maximum elongation (l_max_) corresponds to the maximum strain (ε_max_) experienced by the sensitive element during the trial. The output data given by the tensile machine (i.e., ε, time, applied force and elongation) were collected by a personal computer at a sampling frequency of 10 Hz, whereas the FBG output data were collected by the fiber optic interrogator at a sampling frequency of 100 Hz. The whole process was executed 10 times in order to evaluate the repeatability of the response. All the data were exported and analyzed in MATLAB^®^ (MathWorks^®^ Inc., Natick, MA, USA) environment. In Figure 4, the calibration curve is represented together with its expanded uncertainty. The calibration curve was calculated as the best fitting line considering the average value of Δλ_B_ obtained across the 10 trials over ε. Considering a t-student distribution with nine degrees of freedom and 95% of confidence level [34], it was possible to evaluate the expanded uncertainty as the product of the standard uncertainty and the coverage factor k (i.e., 2.262). The high repeatability of the system was assessed and confirmed by the slight value of the expanded uncertainty (Figure 4). Furthermore, the sensitivity (S_ε_), whose value is 0.20 nm∙mε^−1^, was calculated as the slope of the calibration curve. The correlation coefficient (R^2^) was then evaluated. Its high value (i.e., >0.99) confirms that the behavior of the experimental data agrees properly with the linear model. 

### 2.2. Wearable Device

**Figure 5:**
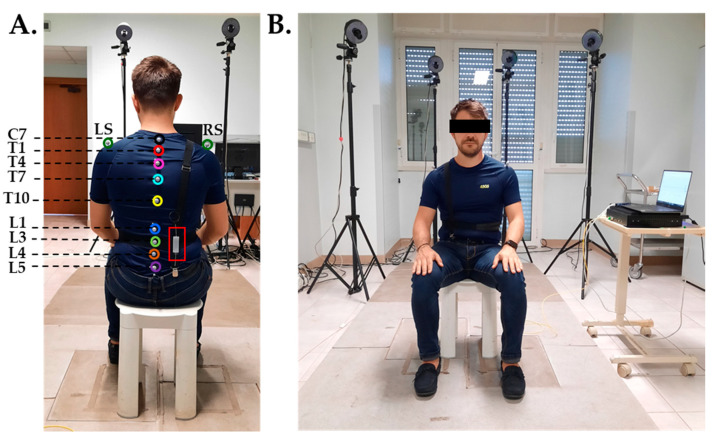
Experimental set up. (**A**). Back view showing the posterior part of the wearable device and the positioning of the flexible sensor (red rectangle) and the photo-reflective markers; (**B**). Frontal view showing the anterior part of the wearable device, the MoCap cameras, the optical interrogator and the laptop.

The proposed wearable device consists of two parts: a flexible, FBG-based sensitive element (described in the previous paragraphs) and an elastic wearable structure. Such a structure is a wearable support composed by two elastic bands stitched orthogonally together by hand. The first band was designed to be worn on the worker’s right shoulder and solidly anchored at the subject’s garments by means of two clips. The second band works like an elastic belt that, once secured with some Velcro^®^ stripes around the subject’s waist, ensures the adherence of the system to the back. The system is adjustable in length, so that it can be worn by subjects with different anthropometric characteristics. The flexible sensing element is fixed upon the back part of the structure (where the two elastic bands cross) with a double-sided adhesive tape for fabrics to be compliant with the physiological lumbar curvature. The proposed wearable system is shown in Figure 5A,B.

## 3. Feasibility Assessment for Monitoring Low Back Movements

Experimental trials were carried out on a group of volunteers to investigate the ability of the proposed wearable device to monitor the low back F/E movements. Volunteers were called to execute a series of F/E in presence of a MoCap system (Smart-D, BTS Bioengineering Corp., Milan, Italy) with IR-photo-reflective passive markers while wearing the wearable. The MoCap was used to record marker’s trajectories, thus further allowing the inter-marker distances analysis and for calculating lumbar angle. 

### 3.1. Population and Experimental Design

Four healthy volunteers (two males and two females) with no history of back disorders were enrolled. The main population characteristics, expressed as mean ± standard deviation, are: age of 28.4 ± 0.5 years old, height of 175.2 ± 4.4 cm, body mass 67 ± 11.7 kg, and chest circumference 94.4 ± 9.5 cm. Each subject wore the elastic structure over a tight t-shirt and was invited to sit on a stool placed at the center of the four-camera MoCap recording area (about 3 m^3^ of calibrated volume) and maintain a straight posture. In line with the protocol proposed in [35] by Chockalingam et al., 11 photo-reflective passive markers with a diameter of 18 mm were positioned on specific body landmarks (i.e., C7, T1, T4, T7, T10, L1, L3, L4, L5, right and left shoulder) by means of a bi-adhesive tape (see Figure 5A). The FBG-based flexible sensor was then fixed with bi-adhesive tape for textiles upon the elastic wearable structure, in correspondence with the lumbar area between the subject’s L1 and L5 lumbar vertebrae, as shown in Figure 5A. The volunteer was instructed to follow the protocol that consisted in executing four consecutive back flexions followed by four consecutive extensions two times, for an overall of sixteen F/E movements per trial. Each volunteer repeated the protocol twice; a total amount of 8 trials was collected. During the trials, the outputs of both the wearable system and the MoCap system were acquired. An optical spectrum interrogator (si255, Micron Optics Inc., Atlanta, GA, USA) was used to collect the FBG outputs at a sampling rate of 100 Hz, while the positions in time of the photo-reflective markers were collected by the MoCap at the sampling frequency of 60 Hz and processed with a dedicated software (i.e., OEP-Smart, BTS Bioengineering Corp., Milan, Italy) to obtain the trajectories of the F/E movements. The entire experimental set-up is shown in Figure 5B.

### 3.2. Data Analysis 

**Figure 6:**
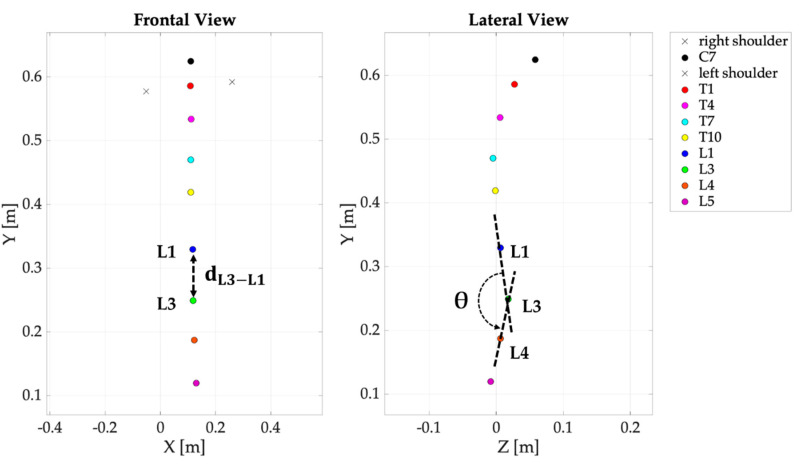
The distance $d_{L3-L1}$ (**left image**) and the lumbar angle θ (**right image**) retrieved from markers’ trajectories.

Per each trial, from markers’ trajectories the distance between markers L1 and L3 ($d_{L3-L1}$, see Figure 6) was calculated as in the following formula:

(3) $$d_{L3-L1}=\sqrt{\left({\left(x_{L3}-x_{L1}\right)}^{2}+{\left(y_{L3}-y_{L1}\right)}^{2}\right)}$$

where $x_{L3}$ and $x_{L1}$ are the *x*-axis coordinates of L3 and L1, respectively and $y_{L3}$ and $y_{L1}$ the *y*-axis coordinates. The Δ$d_{L3-L1}$ was then calculated as

(4) $${\Delta d}_{L3-L1}={\;d}_{L3-L1}-d_{L3-L1}{|}_{t=0}$$

This value allowed us to quantify the relative distance between L1 and L3 (expressed in cm) during F/E movements.

Considering one trial, the first maximum peaks recognized both on the Δλ_B_ and on the Δ$d_{L3-L1}$ signals were used to synchronize the wearable device and the MoCap. Then, the Δλ_B_ and Δ$d_{L3-L1}$ data recorded during the first flexion movement were used to calibrate the wearable device output for reconstructing the L1-L3 displacements from Δλ_B_ (to obtain Δ$d_{\Delta \lambda }$).

Since the linear relationship between the ε and the Δλ_B_ (see Figure 4), a least-squares linear regression was carried out to accomplish this task, considering the ${\Delta d}_{L3-L1}$ as predictor variables and Δλ_B_ as response variables as in Equation (5),

y = α + βx + ζ(5)

where α is the y-intercept (fixed at 0), β the slope (or regression coefficient), and ζ the error term. 

To quantify the goodness of regression, the coefficient of determination (R^2^) was calculated. 

The obtained calibration coefficient β was then applied to the whole signal Δλ_B_ to obtain Δd_Δλ_B__ signal as in the following equation:

Δd_Δ__λ_B__ = β Δλ_B_ (6)

To quantify the difference between the distance ${\Delta d}_{L3-L1}$ and the reconstructed distance Δd_Δ__λ__B_ the Mean Absolute Error (MAE_Δd_) coefficient was calculated as in the following equation:

(7) $${\mathrm{MAE}}_{\Delta d}=\frac{\sum_{i\;=1}^{N}\left|{\Delta d}_{\Delta \lambda_{B}}-{\Delta d}_{L3-L1}\right|}{N}$$

Additionally, the lumbar angle (θ) was calculated considering the trajectories of L1, L3 and L4 as shown in Figure 6. In particular, θ was obtained as the angle among two vectors ($\vec{L1L3}\;$ and $\vec{L3L4}$) at each instant:

(8) $$\theta =\cos^{-1}\left(\frac{\vec{L1L3}\;\cdot \vec{L3L4}}{||\vec{L1L3}||\cdot ||\vec{L3L4}||}\right)$$

### 3.3. Results

**Figure 7:**
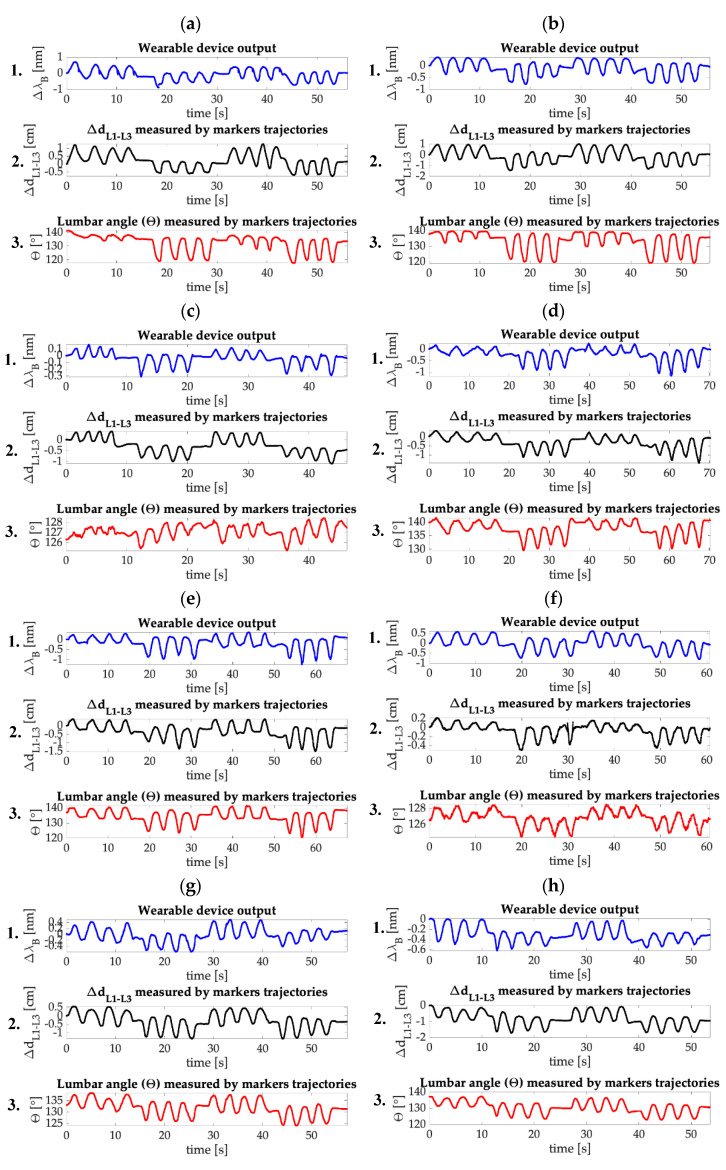
(**1**) The wearable output (Δλ_B_), (**2**) the distance between L1 and L3 (Δ$d_{L3-L1}$) and (**3**) the lumbar angle (θ) trends obtained per each trial. (**a**): Trial 1; (**b**): Trial 2; (**c**): Trial 3; (**d**): Trial 4; (**e**): Trial 5; (**f**): Trial 6; (**g**): Trial 7; (**h**): Trial 8.

In Figure 7 the trends in time of the wearable output (Δλ_B_), the distance between L1 and L3 (Δ$d_{L3-L1}$), and the lumbar angle (θ) evaluated for each trial are reported. It is possible to observe that the FBG and the reference system outputs show great agreement. In fact, as visible from the Δλ_B_ and the Δ$d_{L3-L1}$ trends, the wearable device was able to follow the movements performed by the volunteers for the entire duration of the trials. In particular, the system succeeded in following the protocol even during the pauses between each set of flexions and extensions and during the execution of minimal movements (see Figure 7f) which confirms the high sensitivity of the flexible sensor. The trends in time of θ are used as a further reference to evaluate the amplitude of the F/E movements performed during the execution of the protocol. Moreover, F/E are clearly distinguishable: flexions are defined as the signal peaks (which are the portions of the signal between two consecutive minimum points starting from the first recorded value), while the extensions are defined as the signal valleys (which are the signals included between two consecutive maximum points starting from the first recorded value). For every trial, eight flexions and extensions can be counted, with a total amount of sixteen F/E movements, as expected. The widest ∆λ_B_ excursion that occurs during the trials is about 2 nm. It is worth noting that such strain condition was widely evaluated during the mechanical characterization of the flexible sensor (see Figure 4).

Here below, Table 1 summarizes the β regression and the R^2^ coefficients related to the regression procedures. As shown in the table, the β differs trial by trial from 0.32 cm·nm^−1^ to 2.78 cm nm^−1^; all the R^2^ values denote moderate to good quality of regression.

Table 2 reports the range of ${\Delta d}_{L3-L1}$ calculated from data recorded by the MoCap together with the MAE_Δd_. The maximum value of MAE_Δd_ was 0.33 cm (~16% on the ${\Delta d}_{L3-L1}$ amplitude of 2.02 cm, as in Figure 7a).

**Figure 8:**
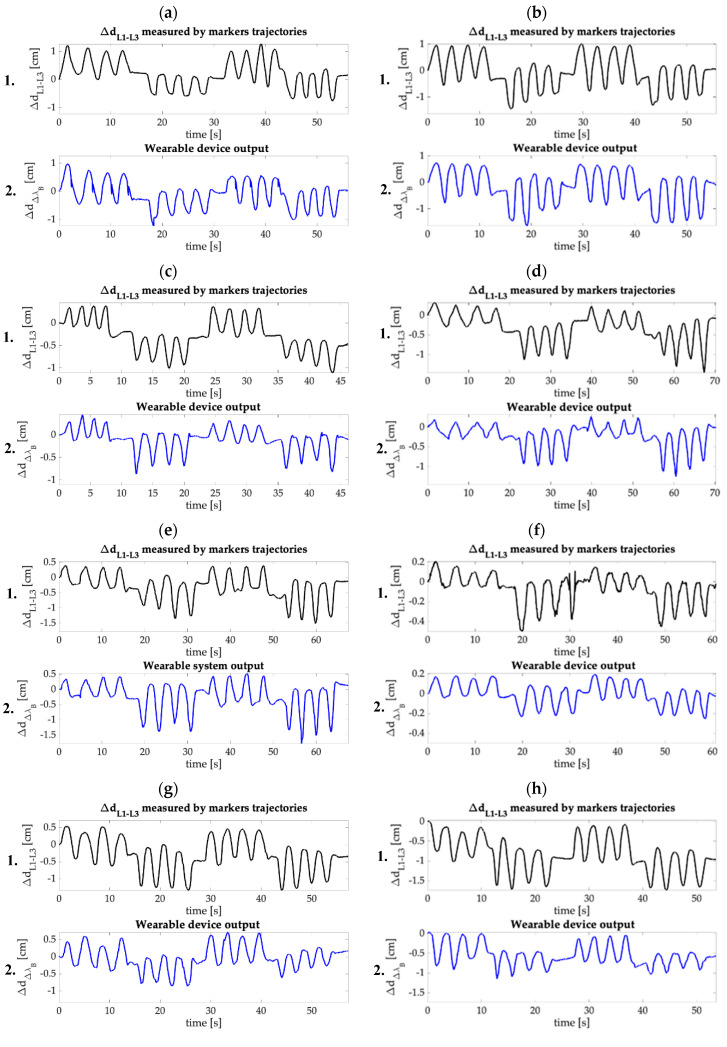
(**1**) The distance between L1 and L3 evaluated by the MoCap (Δ$d_{L3-L1}$) and (**2**) the reconstructed distance (${\Delta d}_{\Delta \lambda B}$ ) obtained per each trial. (**a**): Trial 1; (**b**): Trial 2; (**c**): Trial 3; (**d**): Trial 4; (**e**): Trial 5; (**f**): Trial 6; (**g**): Trial 7; (**h**): Trial 8.

In Figure 8, the distance between L1 and L3 evaluated by the MoCap system (Δ$d_{L3-L1}$) and the reconstructed distance (Δd_Δλ_B__) are shown for each trial. Once again, it is possible to appreciate the concordance of the two signals over time. Also, in this case F/E movements are clearly distinguishable, as well as the minimal movements and the pauses performed between each set of flexions and extensions.

## 4. Discussion and Conclusions

A prompt detection of unnatural and uncomfortable postures in stationary employees by monitoring spinal ROMs can help to avoid the occurrence of LBP in the working population. LBP represents a relevant socio-economic burden as is responsible for the exploitation of the National Health Service and absenteeism in workplaces [6,7]. Many contactless and contact-based devices for ROMs detection are available on the market [11,12,13,14,15,16,17,18], but most of them are bulky, invasive or uneasy to wear during the working hours. In the last decades, the advantages brought by FOSs (i.e., flexibility, lightness, handling and multiplexing capability [22,23]) led to the increasing use of FOSs-based wearable devices [22,23,24]. To date, no wearables based on FBG technology for lumbar ROMs detection are available in the literature.

In this paper, a new smart wearable device to detect low F/E movements, composed by a comfortable elastic structure and a flexible FBG-based sensor is presented. Both the elastic structure and the flexible sensor are custom-made. The sensor was produced by housing a commercial FBG into a rectangular bi-component silicone matrix. The flexible element response to strain was assessed: a linear response to strain with S_ε_ = 0.20 nm∙mε^−1^ have been found. Then, the feasibility assessment of the wearable device in measuring F/E in the sagittal body plane was experimentally executed on a small population of volunteers, in presence of the MoCap system as gold standard. The lumbar ROMs was evaluated for each trial from the MoCap output in terms of Δ$d_{L3-L1}$ and θ. For every trial, the Δλ_B_ and Δd_Δλ_B__ obtained by the FBG have been compared with the Δ$d_{L3-L1}$ and θ, showing in both the cases good accordance in signal trends (see Figure 7 and Figure 8). Flexions and extensions are clearly distinguishable, and the system is perfectly able to follow the performed movements (including pauses and minimal motions) during the entire duration of the trials. β and R^2^ values were calculated to evaluate the sensitivity and the linearity of the wearable system, while MAE_Δd_ values were calculated to investigate the agreement between Δd_ΔλB_ and Δd _L3-L1_ (see Table 1 and Table 2). As visible, the β values range from 0.32 cm·nm^−1^ to 2.78 cm·nm^−1^. Such a dispersion might be attributable to two causes: the sensor manufacturing process (in fact, as demonstrated by Tang et al. in [36], the sensitivity of FBG-based sensors strictly depends on the housing material shape and stiffness) and the intra and inter subject variability of the sensor positioning (i.e., change in position of the sensor caused by the movements performed by a single volunteer during the trials execution, and the sensor different positioning between different subjects which is determined by the dissimilar anthropometric characteristics). In our experimental scenario, the wide variations in β values can be ascribable to the second cause, as the same sensor was exploited during the entire experimental protocol. Additionally, the lowest value of R^2^ (i.e., 0.66) might be attributable to the deviation of the device behavior from the linear one caused by the sliding of the wearable from its initial placement while performing the experimental routines. This phenomenon can justify the quite wide ranges of values found for MAE_Δd_, as well. Consequently, an optimization of the integration and coupling system of the sensor, together with the calibration process to be performed at the beginning of each trial, will help minimize these undesired effects.

In the clinical practice, one of the most reliable and accurate technique to detect lumbar ROMs is the use of radiography [13,14]. In fact, intervertebral angles can be clearly identifiable by evaluating the radiographic images of subjects’ back in F/E postures. Unfortunately, the disadvantages brought by such practice are several, as it is time consuming and extremely invasive due to repeated X-ray exposures. Therefore, in the last decades, clinicians have been moving towards the use of ever safer, less invasive and immediate techniques and devices. 

In the literature, lumbar ROMs has been detected using a wide range of devices based on non-wearable (i.e., goniometers [12] and MoCap systems [14]) and wearable (i.e., MEMS accelerometers and piezoresistive textiles [16], strain gauge sensors [17], and FOSs-based systems [18,19,20]) technologies. 

Focusing on non-wearable solutions, goniometers [12] are intuitive and easy-to-use tools for ROM detection that expect direct contact with the subjects. However, the quality of the measurements strictly depends on the operator’s skills and experience, since the procedure is carried out manually. Furthermore, goniometers do not guarantee information regarding the lumbar kinematic pattern over the working hours. On the contrary, MoCap systems [14] are undoubtedly excellent devices for the dynamic monitoring of lumbar F/E allowing the subject to move freely within the calibrated volume. Unfortunately, their high costs and the need of structured environments (i.e., infrared cameras and passive photo-reflective markers on specifics body landmarks) make this technology unusable in real working scenarios. Otherwise, the proposed wearable device enables the continuous detection of the lumbar kinematic. Moreover, it is easy to wear, unobtrusive, and does not require any other items to be applied (i.e., photo-reflective markers) and/or structured environments. 

Focusing on wearable solutions, different technologies have been proposed. A hybrid system based on piezoresistive and MEMS technology integrated into a rowing [16] and a sensing solution composed by two flexible stripes embedding 12 strain gauge (Epionics SPINE system) [17] were presented. In both the studies a MoCap system was used as a benchmark to assess the capability in detecting F/E. The system in [16] was tested only on a single volunteer during six trials of F/E showing a 2% error in length estimation when compared with a MoCap system, while the one in [17] was tested on 20 volunteers to monitor lumbar ROMs during standing, walking, running, and sitting, showing moderate agreement with the benchmark. Both the systems are bulky due to the presence of multiple cables and additional tools (i.e., MEMS placed on the sacrum and the T12 thoracic vertebra [16], and a portable storage unit fixed to a belt [17]). These limits do not permit long-term use in the scenario of interest. Despite our system reaching MAE values higher than 2% and being assessed in sitting positions, it is unobtrusive and compliant with the natural spinal curvature, so improving the system acceptability for the users. In addition, the optical signal is not affected by noises and electromagnetic interferences permitting a large-scale employment even in challenging and harsh environments (e.g., in the presence of strong electromagnetic fields). 

Recently, FOSs technology have gained attention for low back F/E monitoring. In [18] and [19], the ShapeTape commercial device was equipped with several arrays of fiber optic sensors to detect spinal curvature through the measurement of transmitted light intensity. Both these solutions were tested on 13 and 26 volunteers, respectively, using a MoCap system as a benchmark. Results showed a good agreement in measuring the spinal curvatures related to seated postures. However, such systems require to be fixed on the workers’ skin, making their use impractical in workplaces. On the contrary, a comfortable, commercial tight shirt integrated with FOSs was proposed in [20] making a step forward in terms of lightness and wearability. Its feasibility was assessed on nine volunteers performing F/E movements while sitting and in presence of a MoCap system. Such wearable showed good capability in detecting ROMs, but its integration into a garment limits its usability on a wide range of population with different anthropometric characteristics and gender. Otherwise, our wearable device consists of two parts to be easily worn on everyday clothes by both males and females with different body shapes and sizes. 

In conclusion, the proposed wearable device is the first one based on FBG technology for detecting and monitoring low back F/E movements. The strength of the presented wearable device relies on the high sensitivity of the flexible sensor that permits a continuous lumbar monitoring over time (even in case of pauses between the F/E movements or minimal motions), as well as the distinction of flexion and extension phases. Moreover, the design of the structure allows the proposed solution to be easily worn over any type of garment, while its lightness and compactness permit the use of the device for the entire working day. Furthermore, thanks to the adjustable elastic suspender, the smart wearable can fit to any body shape and size. 

The proposed device lays the basis for the development of FBG-based wearables for workers’ safety monitoring and could carry the research one step forward in the field of the occupational health. 

Lastly, to overcome the large dispersion of MAE_Δd_ and β values, a better and firmer integration of the sensor inside the wearable is foreseen, in order to better adjust and hold the sensor positioning. Additionally, starting from this preliminary prototype, future works will face the creation of a multipoint wearable system capable to monitor the entire spinal ROMs. Exploiting the FBGs multiplexing capability, several gratings will be added into the same fiber, in correspondence with specific spinal landmarks. The use of Neural Networks could be included for the ROMs evaluation. Further tests will be performed to enlarge the sample size and the protocol will be improved and enhanced in order to face realistic working conditions. 

## Figures and Tables

**Table 1:** Calibration coefficient β used to reconstruct Δd_ΔλB_ from Δ_λB_ and R^2^ coefficients resulting from the linear regression between ${\Delta d}_{L3-L1}$ and Δλ_B._

**Trial**	**#1**	**#2**	**#3**	**#4**	**#5**	**#6**	**#7**	**#8**
β [cm·nm^−1^]	1.38	2.10	2.78	1.08	1.43	0.32	1.45	1.84
R^2^	0.92	0.78	0.89	0.66	0.91	0.93	0.88	0.88

**Table 2:** MAE_Δd_ values used to quantify the difference between the distance ${\Delta d}_{L3-L1}$ and the reconstructed distance Δd_Δ__λ__B_.

**Trial**	**#1**	**#2**	**#3**	**#4**	**#5**	**#6**	**#7**	**#8**
MAE_Δd_ [cm]	0.33	0.18	0.21	0.14	0.14	0.07	0.28	0.29
